# Metabolically healthy obesity, transition to unhealthy phenotypes, and type 2 diabetes in 0.5 million Chinese adults: the China Kadoorie Biobank

**DOI:** 10.1530/EJE-21-0743

**Published:** 2021-12-07

**Authors:** Zimin Song, Meng Gao, Jun Lv, Canqing Yu, Yu Guo, Zheng Bian, Yuxia Wei, Ling Yang, Huaidong Du, Yiping Chen, Jianqiang Zhang, Jvying Yao, Junshi Chen, Zhengming Chen, Tao Huang, Liming Li, the China Kadoorie Biobank (CKB) Collaborative Group

**Affiliations:** 1 Department of Epidemiology and Biostatistics, School of Public Health, Peking University Health Science Center, Beijing, China; 2 Key Laboratory of Molecular Cardiovascular Sciences (Peking University), Ministry of Education, Beijing, China; 3 Peking University Institute of Environmental Medicine, Beijing, China; 4 Chinese Academy of Medical Sciences, Beijing, China; 5 Medical Research Council Population Health Research Unit at the University of Oxford, Oxford, UK; 6 Clinical Trial Service Unit & Epidemiological Studies Unit (CTSU), Nuffield Department of Population Health, University of Oxford, Oxford, UK; 7 Zhouquan Town Health Center, Tongxiang, Zhejiang, China; 8 Gaoqiao Town Health Center, Tongxiang, Zhejiang, China; 9 China National Center for Food Safety Risk Assessment, Beijing, China

## Abstract

**Objectives:**

To prospectively assess the association of metabolic health status and its transition with incident diabetes risk across BMI categories.

**Design:**

Cohort study based on the China Kadoorie Biobank (CKB).

**Methods:**

The CKB study enrolled 512 715 adults aged 30–79 years from ten diverse areas in China during 2004–2008. After exclusion, 432 763 participants were cross-classified by BMI categories and the metabolic status was followed up for incident diabetes disease. The changes in BMI and metabolic health status were defined from baseline to the second resurvey.

**Results:**

Type 2 diabetes risk is higher for metabolically healthy obese (MHO) subjects than metabolically healthy normal weight (MHN) individuals (HR: 3.97, 95% CI: 3.64–3.66), and it is highest for those affected by metabolically unhealthy obese (MUO) (HR: 6.47, 95% CI: 6.17–6.79). About 15.26% of participants with MHN converted to metabolically healthy overweight or obesity (MHOO), whereas 48.40% of MHOO remained unconverted throughout the follow-up. In obese or overweight people, the conversion from metabolically healthy to unhealthy might increase the chances of developing diabetes as compared to those with a stable metabolic healthy state (HR: 3.70, 95% CI: 2.99–4.59), while those with persistent metabolic disorders are most likely to have diabetes (HR: 8.32, 95% CI: 7.08–9.78).

**Conclusions:**

Metabolic healthy is a transient state, and individuals converted from metabolically healthy status to unhealthy phenotypes across all BMI categories might raise the risk of diabetes.

## Introduction

The worldwide burden of diabetes is enormous and increasing sharply, estimated to be 438 million by 2030 (1). A national survey in China showed that the estimated prevalence of diabetes was 12.8% in 2017 (2). Obesity and related metabolic disorders have been a well-established causal factor for type 2 diabetes mellitus (T2DM) (3, 4). Nevertheless, some obese individuals may not develop metabolic disorders like elevated blood pressure, abnormal lipid profiles, and low insulin sensitivity. This subgroup of obesity displaying a relatively favorable metabolic proﬁle may be referred to as ‘metabolically healthy obesity**’** (MHO) (5, 6). Generally, it is estimated that 3–22% of the general population is affected by MHO (7, 8).

Large-scale epidemiological studies had shown that MHO adults had a substantially increased risk of developing T2DM compared with metabolically healthy normal-weight (MHN) participants among Europeans, Japanese, and Koreans (9, 10, 11, 12), and little previous literature systematically examined the diabetes risks among individuals stratified by metabolic health status across BMI categories in Chinese people (13, 14). Furthermore, it is worth noting that metabolic healthy might be a transient phenotype, and MHO individuals were more likely to develop metabolic risks (15, 16). However, most studies might overlook this issue, and those focused on transition had a small sample size or limited the research population to females (11, 15). Therefore, how the diabetes risk is stratified in this particular population, how metabolic risk factors change in different BMI groups in the long-term follow-up, and how the onset of metabolic disorders affects diabetes risk remain to be elucidated. Given the increasing prevalence of obesity in Chinese adults, a better understanding of the health consequences of MHO phenotypes and their transitions could contribute to public health and clinical practice, as well as promote more efficient diabetes prevention and management strategies.

Therefore, in the present study, we aimed to examine the extent to which the MHO phenotypes, defined using the ATP-III criteria, and their transitions were associated with incident T2DM across BMI categories over the median follow-up of 11.5 years among Chinese adults using data from the China Kadoorie Biobank (CKB).

## Methods

### Study design and participants

CKB was a population-based longitudinal cohort study started in 2004 with 512 715 Chinese adults aged 30–79 years recruited from ten sites (five urban and five rural areas) in China. About 25 239 (5%) participants were randomly invited to the resurvey between August 4, 2013, and September 18, 2014. Detailed information on the study design and data collection in CKB had been reported previously (17, 18). All participants filled out an interviewer-administered electronic questionnaire, providing detailed information on sociodemographic characteristics, medications, lifestyle and dietary factors, and a written informed consent form. Participants with a history of diabetes (*n* = 30 300), stroke (*n* = 8884), coronary heart disease (*n* = 15 472) or cancer (*n* = 2578), as well as those with missing data on plasma glucose (*n* = 8160) or BMI (*n* = 2) were excluded from this study. The Ethical Review Committee of the Chinese Center for Disease Control and Prevention (Beijing, China) and the Oxford Tropical Research Ethics Committee, University of Oxford (UK) approved the study.

### Measurement of covariates and metabolic status

**Figure 1:**
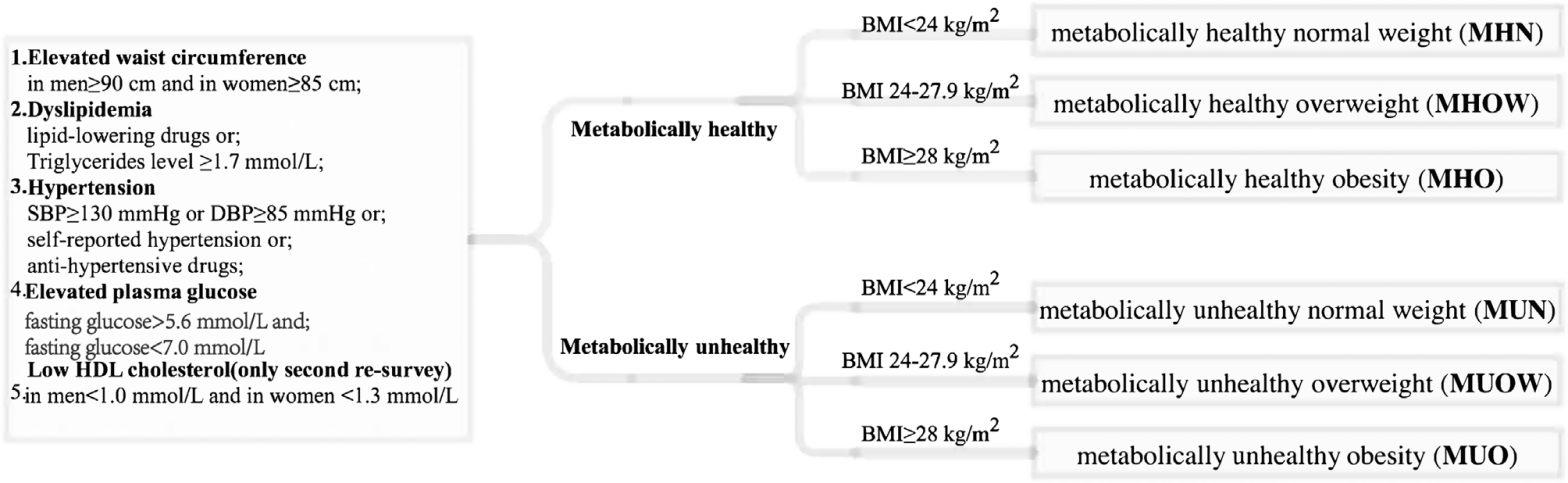
Definition of metabolically healthy obesity and their transition. SBP, systolic blood pressure; DBP, diastolic blood pressure.

Sociodemographic variables (age, sex, education, occupation, marital status, and household income), lifestyle (smoking, alcohol consumption, and physical activity), and dietary factors (fruit, vegetable, and meat intakes) were self-reported at baseline. Height, weight, waist and hip circumferences, and blood pressure were obtained from physical measurements using a standard protocol and validated instruments (19). BMI categories and metabolic status were classified based on Chinese guidelines and a modified definition of the metabolic syndrome described by international harmonized criteria (20, 21, 22). Cross-classification of BMI categories and metabolic status (healthy/unhealthy) created six groups (Fig. 1). As shown in Fig. 1, participants who had two of the four criteria were considered metabolically unhealthy at baseline. We repeated the primary analysis using triglyceride (TG) and HDL-C data from the second resurvey to reduce the impact of dyslipidemia classification errors at baseline, as dyslipidemia was diagnosed based on self-reported use of statins. Therefore, participants who met three or more of the five criteria were rated as metabolically unhealthy in the second resurvey. We also described the transition between different BMI and the metabolic status during follow-up.

### Follow-up for incident diabetes and diabetes mortality

Cases of diabetes and deaths were ascertained by linking disease and mortality surveillance system and the national health insurance claim database, with almost universal coverage across the ten study areas (>98%), supplemented by local residential records and annual active verification. The causes of death were determined primarily by reviewing death certificates, as well as medical records and verbal autopsies using validated instruments. Annual active follow-up was performed by checking against local residential records to confirm survival status and to minimize follow-up losses. More details had been reported elsewhere (23).

All events were coded according to the International Classification of Diseases, 10th Revision (ICD-10) by trained staff blinded to baseline information. Diagnoses of total diabetes are coded according to the International Classification of Diseases, 10th Revision (ICD-10; diabetes: E10–E14). Total diabetes contains insulin-dependent (E10), non-insulin-dependent (E11), malnutrition-related (E12), other specified (E13), and unspecified (E14). The primary outcomes in the present study were type 2 diabetes mellitus (E11) and total diabetes (E10–E14).

### Statistical analysis

Baseline and resurvey sample characteristics are presented as mean (s.d.) or percentage when appropriate. The ANOVA test was used to examine differences between groups in baseline characteristics for continuous variables and the χ^2^ test for dichotomous measures. The Cox proportional hazard regression model with a time-varying exposure was used to examine the relationship between six phenotypes of MHO and their transition associated with the risk of diabetes over the follow-up. Person-time was calculated from the date of birth to diabetes diagnosis, death, or the end of follow-up (Dec 31, 2016), whichever came first. Proportional hazards assumption was tested using Schoenfeld residuals. 95% CIs were estimated by the log-rank test (24). To account for the competing risks of death, competing-risks regression models based on Fine–Gray model were fitted to estimated sub-hazard ratios of MHO to address the potential competing risk problem from mortality (25).

MHN individuals were used as a reference group in the primary analysis. The main analyses were first adjusted for age, sex, study region, and then further for education, household income, marital status, smoking status, alcohol intake, physical activity, fruit, vegetable and red meat intakes, and family history of diabetes. In categorical analyses, participants were divided into six groups according to MHO phenotypes. HRs were calculated relative to the MHN group and plotted against the MHO six phenotypes. To test whether the pattern of association varies across stratifications, we estimated multiplicative interactions by including the product term (exposure × stratification variable) in the models using the likelihood ratio test. During the transition analysis, we used stable MHN during the follow-up as the referent group; HRs were also calculated for MHN to metabolically healthy overweight or obesity (MHOO) group, MHOO throughout the group, MHOO to metabolically unhealthy overweight or obesity (MUOO), and MUOO throughout the group.

A subgroup analysis based on sex or age then allowed us to compare the incidence of T2DM among the different groups. Sensitivity analyses were undertaken to assess the impact of excluding cases that had been smokers or occurred in the first 2 years of follow-up or using waist–hip ratio instead of waist circumference to demonstrate the robustness of our results. We also investigated whether results were similar when the metabolic syndrome is defined using the harmonized International Diabetes Federation definition, which excludes waist circumference. Two-sided *P* < 0.05 was considered statistically significant. All statistical analyses were performed using Stata (version 15.0, StataCorp) and SAS version 9.4 (SAS Institute Inc., Cary, NC, USA).

## Results

### Characteristics of the CKB participants

**Figure 2:**
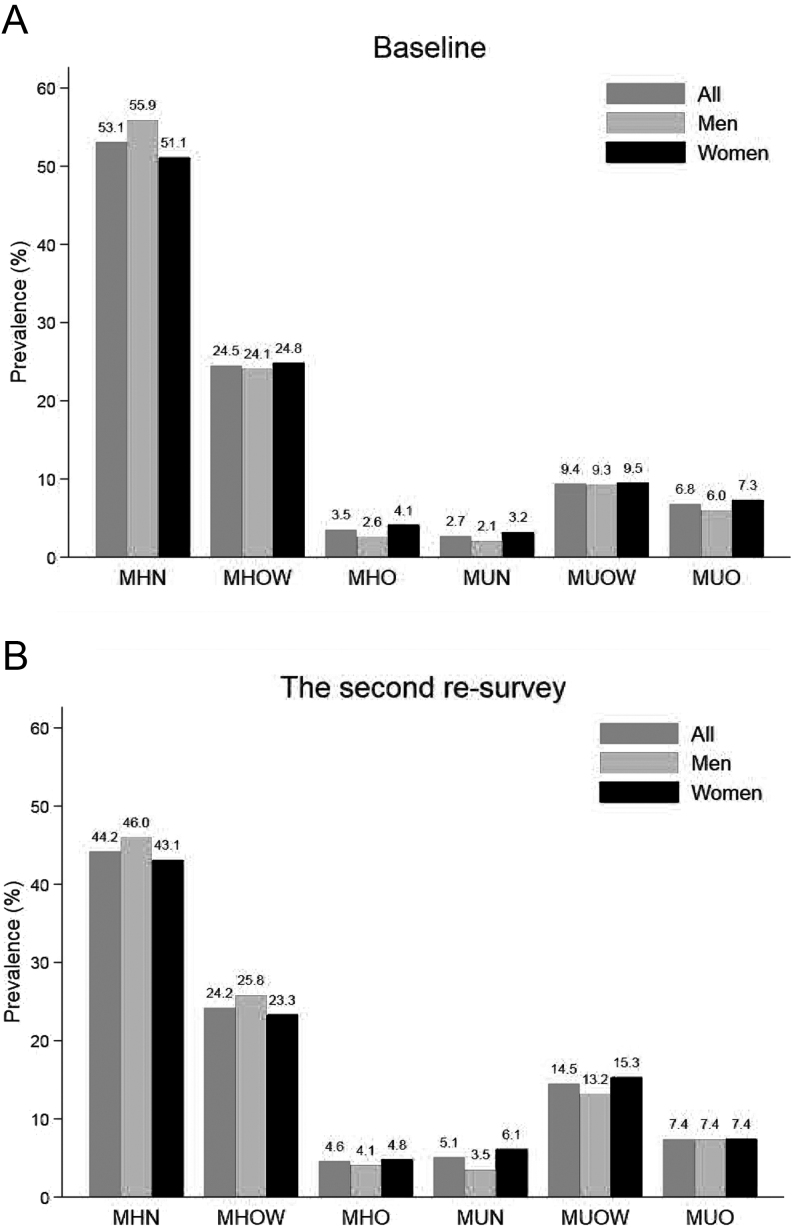
Sex-specific prevalence of MHO at baseline and second resurvey. ^†^Prevalence is standardized for age and region. MHN, metabolically healthy normal weight; MHO, metabolically healthy obesity; MHOW, metabolically healthy overweight; MUN, metabolically unhealthy normal weight; MUO, metabolically unhealthy obesity; MUOW, metabolically unhealthy overweight.

Of 432 763 participants in this analysis, the mean age at baseline was 51.0 (10.4) years, mean BMI was 23.8 (3.1) kg/m^2^, 177 317 (41.0%) were men, and 188 072 (43.5%) resided in urban areas (Table 1). Also, 4571 (1.06%) of 432 763 participants failed to follow up, and the rate was not significantly different between the MHO groups after adjusted age, sex, and region (*P* for trend = 0.291) (data not shown). More than half of the participants (53.4%) had metabolically healthy normal weight, and only 3.5% were MHO, in which more women than men (4.1% vs 2.6%) (Fig. 2 and Supplementary Fig. 1, see section on supplementary materials given at the end of this article). The prevalence of participants with metabolically unhealthy normal weight (MUN), metabolically unhealthy overweight (MUOW), and metabolically unhealthy obesity (MUO) was 2.7, 9.4, and 6.8%, respectively (Fig. 2). Mean age and SBP were higher among metabolically unhealthy individuals across all BMI categories than their healthy counterparts. In other words, individuals with MHO were more likely to be female, younger, living in rural areas, have less physical activity, and have more meat intake than MHN.

**Table 1:** Baseline and the second resurvey characteristics of the study population. Data are presented as mean ± S.D. for continuous variables and percentage for categorical variables.

Characteristics^‡^	Baseline							Second resurvey						
	All	Metabolically healthy			Metabolically unhealthy			All	Metabolically healthy			Metabolically unhealthy		
		Normal weight	Overweight	Obese	Normal weight	Overweight	Obese		Normal weight	Overweight	Obese	Normal weight	Overweight	Obese
No. of participants	432 763	229 721	106 242	14 987	11 820	40 659	29 334	14 894	6579	3612	679	754	2155	1115
Demographic factors														
Age (years)	51.0 (10.4)	50.6 (10.6)	49.7 (9.6)	48.1 (9.1)	57.0 (10.2)	54.8 (10.1)	53.0 (10.0)	57.5 (9.7)	58.0 (10.0)	56.1 (9.3)	56.0 (9.4)	61.1 (9.9)	58.0 (9.4)	57.0 (9.3)
Male (%)	41.0	43.0	39.8	29.9	33.9	41.7	37.1	37.5	39.0	39.6	33.9	26.5	34.4	37.7
Urban (%)	43.5	37.7	48.9	53.1	42.0	50.5	54.8	38.1	33.6	43.6	44.7	33.4	40.8	40.2
Socioeconomic factors														
Middle school or higher (%)	50.3	50.6	51.2	49.5	49.3	49.3	46.9	48.3	48.4	48.9	44.1	51.3	47.7	47.4
Household income≥20 000 yuan/year (%)	43.1	41.7	43.9	43.2	44.4	46.8	44.3	78.5	78.1	79.7	77.5	75.9	79.1	78.7
Married (%)	91.3	90.6	92.4	92.6	90.3	92.1	91.8	89.2	88.6	89.8	89.3	89.8	89.6	89.8
Lifestyle factors														
Current smoker (%)	29.1	30.7	27.0	27.2	29.0	27.6	26.6	23.5	25.1	21.3	20.0	26.3	22.3	23.7
Weekly drinker (%)	15.3	15.2	14.7	14.0	18.4	16.6	15.6	12.7	12.9	12.1	12.0	11.8	12.9	13.7
Physical activity (MET-h/day)	21.8 (13.9)	22.4 (14.1)	21.7 (13.8)	20.8 (13.0)	21.7 (14.2)	20.8 (13.3)	19.9 (12.9)	19.9 (14.0)	20.5 (14.6)	20.3 (13.7)	20.0 (13.4)	19.0 (13.1)	18.6 (13.3)	18.1 (13.1)
Meat intake (days/week)	3.7 (2.5)	3.6 (2.5)	3.8 (2.6)	3.8 (2.6)	3.6 (2.5)	3.8 (2.6)	3.8 (2.6)	4.2 (2.7)	4.2 (2.8)	4.3 (2.7)	4.3 (2.7)	4.1 (2.9)	4.2 (2.8)	4.2 (2.7)
Vegetable intake (days/week)	6.8 (0.8)	6.8 (0.8)	6.9 (0.7)	6.8 (0.7)	6.8 (0.9)	6.8 (0.7)	6.9 (0.6)	6.9 (0.7)	6.9 (0.7)	6.9 (0.5)	6.9 (0.7)	6.9 (0.8)	6.9 (0.7)	6.9 (0.7)
Fruit intake (days/week)	2.6 (2.5)	2.5 (2.4)	2.7 (2.5)	2.7 (2.7)	2.4 (2.4)	2.6 (2.6)	2.6 (2.7)	3.6 (2.7)	3.6 (2.7)	3.7 (2.7)	3.7 (2.6)	3.6 (2.7)	3.6 (2.7)	3.7 (2.7)
Physical measurements														
BMI (kg/m^2^)	23.8 (3.1)	21.6 (1.5)	25.4 (1.1)	29.4 (1.6)	22.2 (1.4)	26.1 (1.1)	30.1 (2.0)	24.3 (3.1)	21.7 (1.4)	25.5 (1.1)	29.7 (1.9)	22.5 (1.2)	26.0 (1.1)	30.1 (2.0)
Waist–hip ratio	80.5 (9.2)	74.7 (5.9)	83.3 (5.5)	91.5 (7.4)	79.9 (8.4)	89.9 (5.2)	96.2 (6.7)	84.4 (9.2)	77.7 (6.0)	86.7 (5.7)	96.4 (7.0)	83.3 (6.3)	90.6 (5.1)	98.4 (7.2)
SBP (mmHg)	130.4 (20.8)	125.8 (19.6)	129.6 (19.1)	126.4 (15.1)	142.6 (18.3)	144.1 (18.6)	147.0 (19.1)	134.8 (20.1)	130.3 (19.5)	133.1 (19.2)	137.0 (20.4)	142.7 (17.9)	142.5 (18.7)	144.6 (19.0)
DBP (mmHg)	77.8 (11.1)	75.2 (10.4)	77.7 (10.4)	76.5 (8.8)	83.4 (10.3)	84.8 (10.4)	86.2 (10.8)	78.4 (10.9)	75.4 (10.2)	78.0 (10.2)	81.1 (10.5)	81.7 (10.7)	83.0 (10.5)	85.2 (11.1)
Self-reported conditions (%)														
High waist	23.4	1.2	18.5	68.0	36.9	88.4	98.8	38.7	4.9	44.0	92.6	35.8	85.7	98.7
Hypertension	48.7	37.4	42.9	22.2	97.5	95.4	96.0	60.6	48.3	53.2	60.8	92.1	86.3	88.5
Hyperglycaemia	7.8	3.6	2.8	1.1	60.7	22.2	14.3	3.8	1.9	1.5	0.8	14.4	8.7	10.3
High TG	–	–	–	–	–	–	–	35.3	18.0	19.9	12.3	89.5	81.0	77.0
Low HDL-C	–	–	–	–	–	–	–	36.1	22.0	25.0	13.6	81.6	71.1	69.8

^‡^All variables were adjusted for age, sex, and region except for the number of participants, age, and male (%).

DBP, diastolic blood pressure; MET-h/day, metabolic equivalents of task per hour per day; SBP, systolic blood pressure; TG, triglyceride.

### Incident diabetes risk in different metabolic health status

During a median follow-up of 10.1 years, we documented 14 631 cases of total diabetes and 9033 T2DM. In the same BMI category group, individuals with an unhealthy metabolism are more likely to develop T2DM. In comparison with MHN groups, HRs for MUN individuals were 3.00 (95% CI: 2.74–3.29), 4.49 (95% CI: 4.29–4.70) for MUOW individuals, and 6.47 (95% CI: 6.17–6.79) for MUO individuals (Table 2). In metabolically healthy individuals, being overweight or obese still increases the risk of T2DM. Individuals with metabolically healthy overweight (MHOW) (HR: 2.38, 95% CI: 2.29–2.48) and MHO (HR: 3.97, 95% CI: 3.62–4.36) are much more susceptible to diabetes than MHN individuals. As for total diabetes, this effect was also observed but weakened.

**Table 2:** Adjusted hazard ratios for diabetes and diabetes death by categories of obesity and metabolic health^†^.

	MHN	MHOW	MHO	MUN	MUOW	MUO
Total diabetes						
Cases	4025	3611	769	674	2859	2693
Person-years	2 276 509	1 063 475	1 50 375	110 708	385 032	281 159
HR, sex-adjusted	1.00 (0.97–1.03)	2.08 (2.01–2.15)	3.41 (3.17–3.66)	2.90 (2.68–3.13)	3.98 (3.84–4.13)	5.58 (5.37–5.79)
HR, multivariable-adjusted	1.00 (0.97–1.03)	2.06 (1.99–2.13)	3.43 (3.20–3.69)	2.67 (2.47–2.88)	3.88 (3.74–4.02)	5.52 (5.31–5.73)
Type 2 diabetes						
Cases	2290	2267	460	462	1853	1701
Person-years	2 284 124	1 068 966	151 517	111 864	389 368	285 047
HR, sex-adjusted	1.00 (0.96–1.04)	2.39 (2.29–2.49)	3.84 (3.50–4.21)	3.41 (3.11–3.74)	4.63 (4.43–4.85)	6.50 (6.20–6.82)
HR, multivariable-adjusted	1.00 (0.96–1.04)	2.38 (2.29–2.48)	3.97 (3.62–4.36)	3.00 (2.74–3.29)	4.49 (4.29–4.70)	6.47 (6.17–6.79)
Total diabetes death						
Cases	85	33	4	10	36	28
Person-years	2 292 009	1 077 174	153 062	113 775	396 497	291 649
HR, sex-adjusted	1.00 (0.80–1.25)	1.04 (0.74–1.46)	1.15 (0.43–3.07)	1.37 (0.73–2.56)	1.82 (1.31–2.52)	2.36 (1.62–3.43)
HR, multivariable-adjusted	1.00 (0.80–1.25)	1.12 (0.80–1.58)	1.26 (0.47–3.38)	1.52 (0.81–2.84)	2.01 (1.45–2.78)	2.59 (1.78–3.76)
Type 2 diabetes death						
Cases	45	19	2	4	19	15
Person-years	2 292 009	1 077 174	153 062	113 775	396 497	291 649
HR, sex-adjusted	1.00 (0.74–1.36)	1.11 (0.71–1.75)	1.07 (0.27–4.30)	1.09 (0.41–2.92)	1.88 (1.20–2.94)	2.45 (1.47–4.07)
HR, multivariable-adjusted	1.00 (0.73–1.36)	1.20 (0.77–1.89)	1.18 (0.29–4.75)	1.19 (0.44–3.19)	2.05 (1.31–3.22)	2.73 (1.64–4.55)

^†^Sex-adjusted model: Results were adjusted for sex, age (5 years), and study region. Multivariable-adjusted HRs were adjusted for age (5 years), sex, study region, educational level (primary school or lower and middle school or higher), household income (<20 000 yuan/year or ≥20 000 yuan/year), marital status (married, others), smoking status (current regular smoker, not current regular smoker), alcohol consumption (weekly drinker, not weekly drinker), frequency of fruit intake, frequency of vegetable intake, frequency of meat intake (day/week), family history of diabetes, and physical activity (three groups).

HR, hazard ratio; MHN, metabolically healthy normal weight; MHO, metabolically healthy obesity; MHOW, metabolically healthy overweight; MUN, metabolically unhealthy normal weight; MUO, metabolically unhealthy obesity; MUOW, metabolically unhealthy overweight.

Furthermore, obese and metabolically unhealthy participants simultaneously are at greater risk of T2DM death (HR for MHOW: 2.05, 95% CI: 1.31–3.22; HR for MUO: 2.73, 95% CI: 1.64–4.55). Adjusted for competing risk of other causes of death, there still was a positive correlation between MUO and T2DM mortality (subdistribution HR: 3.54, 95% CI: 1.89–6.60). For total diabetes death, the standard Cox regression model may have also underestimated the actual strength of the association between MHO and total diabetes death (subdistribution HR: 3.32, 95% CI: 2.13–5.18 vs HR: 2.59, 95% CI: 1.78–3.76) (Supplementary Table 4).

**Figure 3:**
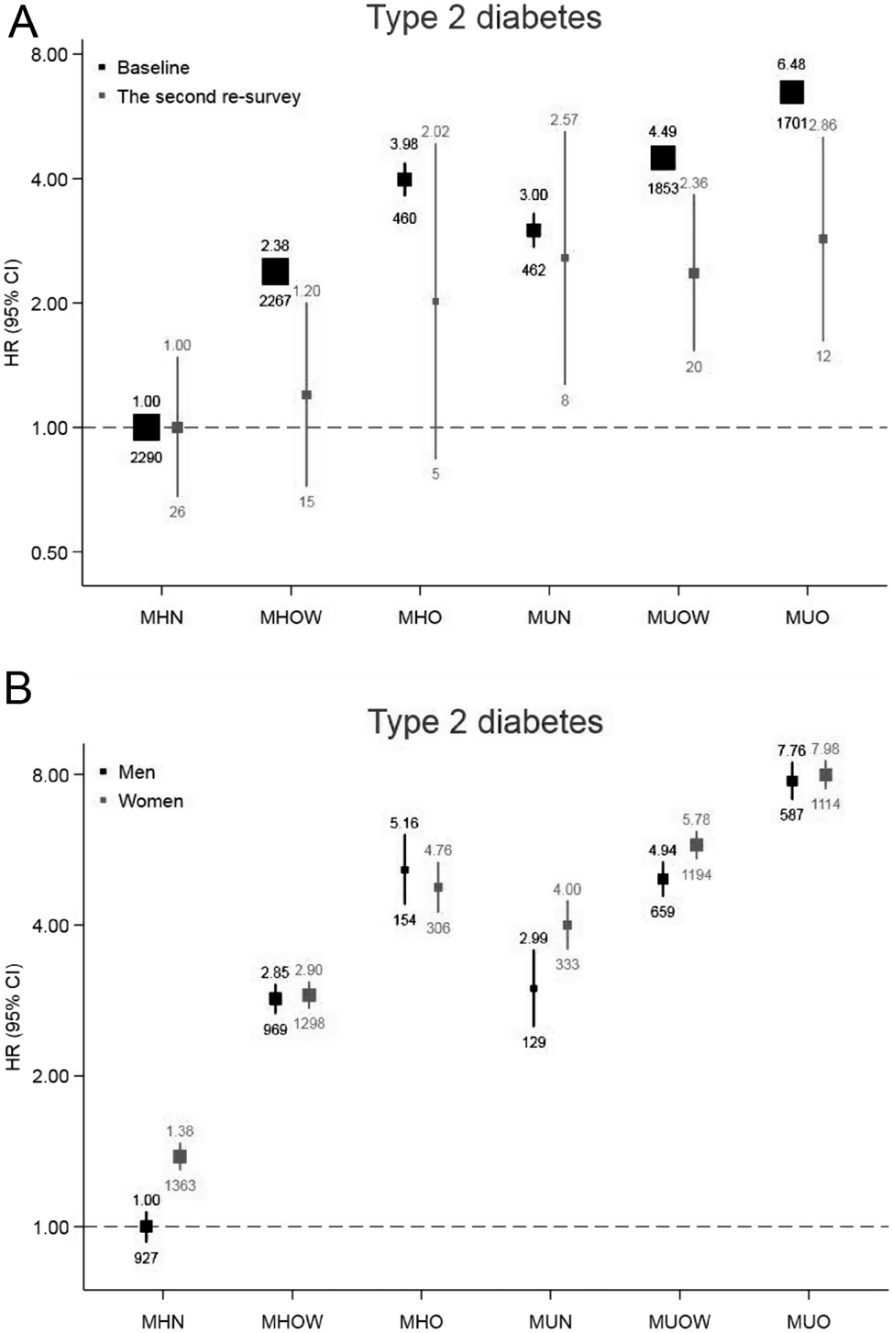
The second resurvey and sex-specific hazard ratios of MHO for type 2 diabetes. ^†^HRs were adjusted for age (5 years), study region, educational level (primary school or lower and middle school or higher), household income (<20 000 yuan/year or ≥20 000 yuan/year), marital status (married, others), smoking status (current regular smoker, not current regular smoker), alcohol consumption (weekly drinker, not weekly drinker), frequency of fruit intake, frequency of vegetable intake, frequency of meat intake (days/week), family history of diabetes and physical activity (three groups). ^‡^HRs are indicated above the squares, and the number of cases in each category is displayed below the squares. Corresponding 95% CIs are plotted as lines. The size of the squares is inversely proportional to the variance of the logarithm of HR. ^*^The relation with MHO differed by sex for type 2 diabetes (*P* for interaction <0.001). MHN, metabolically healthy normal weight; MHO, metabolically healthy obesity; MHOW, metabolically healthy overweight; MUN, metabolically unhealthy normal weight; MUO, metabolically unhealthy obesity; MUOW, metabolically unhealthy overweight; HR, hazard ratio.

On the basis of gender stratification, the six MHO phenotypes posed a slightly higher total diabetes and T2DM risk among women than men, indicating significant modification effects of sex on the associations (*P* for interaction <0.001) (Fig. 3 and Supplementary Fig. 2). When stratified by age groups (30–49 years as the reference group), risks for total diabetes and T2DM gradually increased with aging within each MHO group (*P* for interaction <0.001) (Supplementary Fig. 3). MHO individuals were more prone to develop diabetes than those with normal weight within each age group, while individuals with unhealthy metabolism were most susceptible.

### Sensitivity analyses

In sensitivity analyses, excluding participants who never smoked or cases within the first 2 years after baseline, results were not materially altered. Using a definition of metabolic syndrome that did not contain waist circumference produced enhanced results for MHO; however, with only 0.3% (45 of 14 631) of participants categorized as MHO at baseline, few participants were left to transition to the unhealthy state. We also found a more significant association using waist–hip ratio instead of waist circumference (Supplementary Table 3).

### The transition of metabolic health status and its association with incident diabetes risk

**Figure 4:**
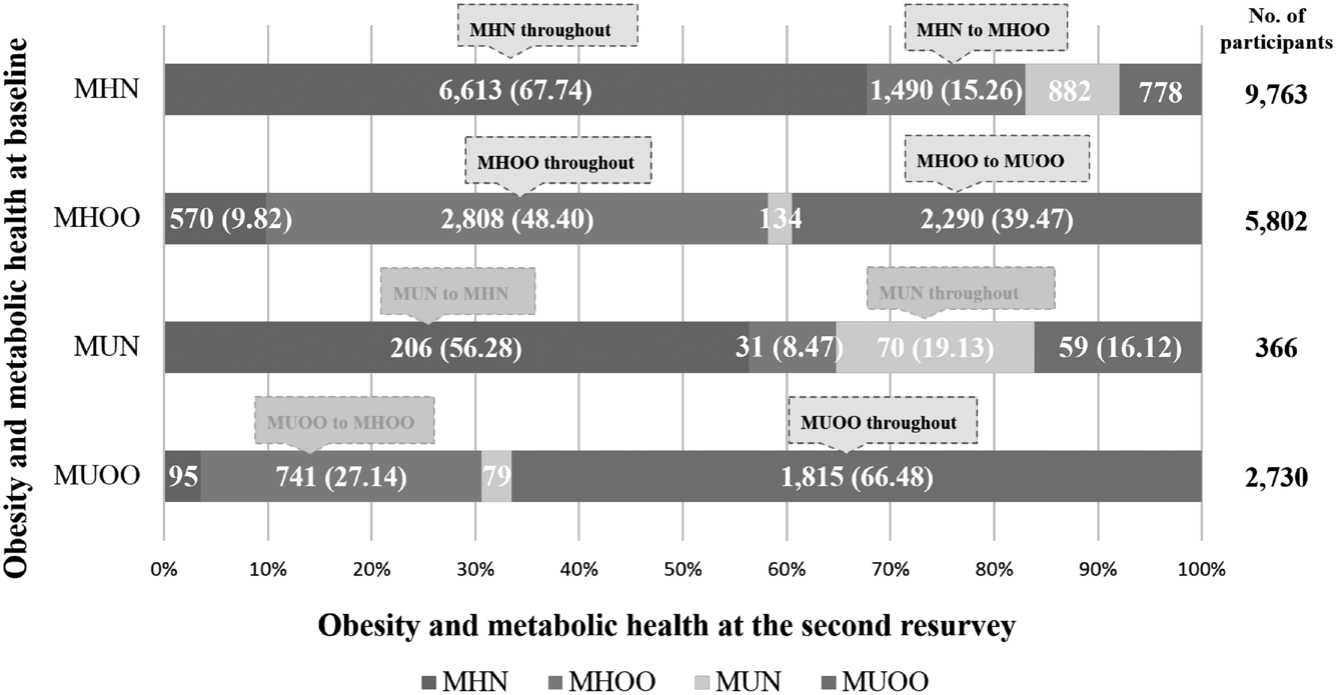
Transition of MHO status from baseline to the second resurvey. MHN, metabolically healthy normal weight; MHOO, metabolically healthy overweight or obesity; MUN, metabolically unhealthy normal weight; MUOO, metabolically unhealthy overweight or obesity.

About 21 890 (5.1%) of baseline participants were randomly invited to conduct the second resurvey. After exclusion criteria, 7355 (39.4%) of 18 661 participants had the transition of metabolic health status in the second resurvey. The sample size of each MHO subgroup for transition is presented in a 6 × 6 contingency table (Supplementary data and Supplementary Table 2). According to the resurvey data, 15.26% of the 9763 participants who had MHN at baseline switched to MHOO phenotypes, while 67.34% did not. Out of 5802 participants with MHOO, 39.47% converted to MUOO and 48.40% failed to convert. However, the majority of MUOO (66.48%) remained unconverted throughout the follow-up (Fig. 4).

**Figure 5:**
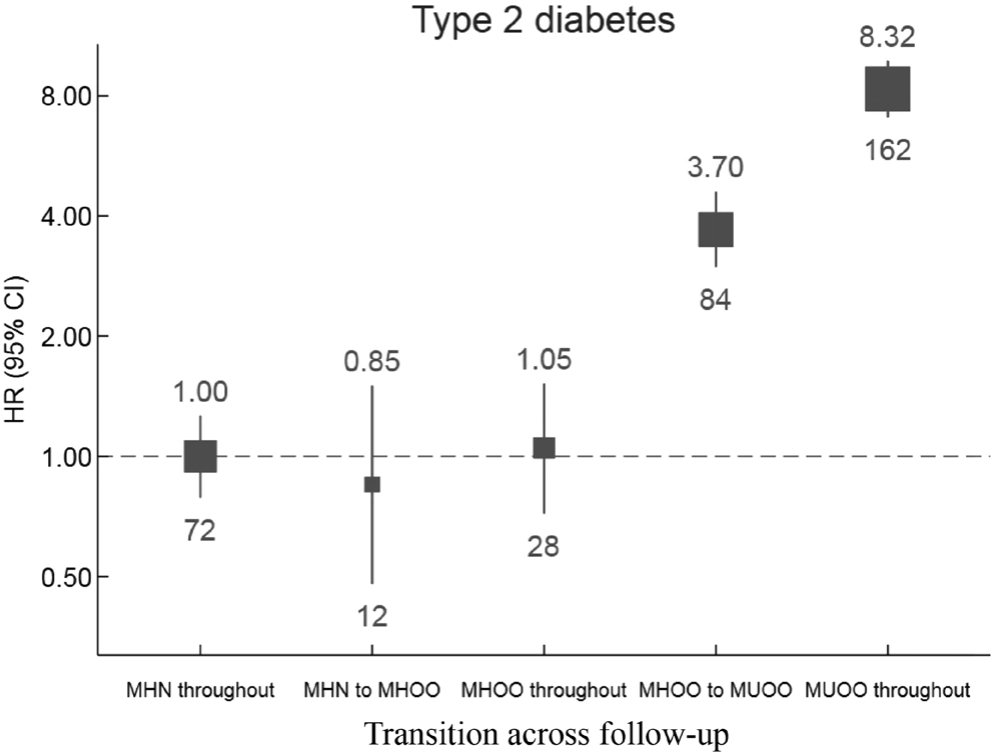
Transition between metabolic status and association with diabetes risk. ^†^HRs were adjusted for age (5 years), sex, study region, educational level (primary school or lower and middle school or higher), household income (<20 000 yuan/year or ≥20 000 yuan/year), marital status (married, others), smoking status (current regular smoker, not current regular smoker), alcohol consumption (weekly drinker, not weekly drinker), frequency of fruit intake, frequency of vegetable intake, frequency of meat intake (days/week), family history of diabetes, and physical activity (three groups). ^‡^HRs are indicated above the squares, and the number of cases in each category is displayed below the squares. Corresponding 95% CIs are plotted as lines. The size of the squares is inversely proportional to the variance of the logarithm of HR. HR, hazard ratio; MHN, metabolically healthy normal weight; MHOO, metabolically healthy overweight or obesity; MUOO, metabolically unhealthy overweight or obesity.

The cumulative incidence of T2DM was higher in participants who changed from metabolic healthy overweight or obesity to MUOO (HR: 3.70, 95% CI: 2.99–4.59) compared to stable MHOO participants (HR: 1.05, 95% CI: 0.72–1.52) but lower than those of stable MUOO participants (HR: 8.32, 95% CI: 7.08–9.78) who had the highest risk among all groups (Fig. 5). For the participants with metabolic healthy normal weight, the risk of T2DM from MHN to obesity was not significant (HR: 0.85, 95% CI: 0.48–1.50) but markedly lower than those who maintained MHOO or converted to MUOO (Fig. 5). Then, we utilized the logistic regression with the sex or multivariable-adjustment models instead of Cox proportional hazards models to analyze the association between MHO transition from the baseline to the second resurvey and diabetes. Compared with participants who maintained MHN, those who were metabolically unhealthy overweight or obesity throughout baseline and the second resurvey had multivariable-adjusted OR for total diabetes of 8.60 (95% CI: 6.78–10.91) and type 2 diabetes of 10.85 (95% CI: 8.04–14.63) (Supplementary data and Supplementary Table 5).

## Discussion

In this prospective study of 432 763 Chinese adults, our findings show that MHO individuals are at an increased risk of incident diabetes compared with MHN individuals over a median of 10.1 years of follow-up, implying that obesity is still the leading risk factor for diabetes and further support should be provided for the need for all individuals to maintain a healthy weight. Besides, metabolically unhealthy individuals had a substantially higher risk of diabetes than individuals without existing metabolic conditions across all BMI groups, supporting strategies to prevent metabolic risk factors irrespective of weight status. Likewise, the transition from healthy to unhealthy overweight or obesity and stable unhealthy overweight or obesity substantially increased the risk of incident diabetes.

Our findings in this prospective cohort study are in line with previous results in Western populations (12, 15). The Whitehall II study among 7122 participants (69.7% men), aged 39–63 years, showed that MHO subjects were at increased risk for type 2 diabetes (HR: 3.25, 95% CI: 2.32–4.54) compared with MHN individuals over the median follow-up of 17.4 years (12). The hazard risk was relatively lower than in our study of 0.5 million Chinese adults (HR: 3.97). Similarly, a meta-analysis of three prospective cohort studies including 134 667 East Asian subjects showed that MHO adults had a distinctly higher risk of developing type 2 diabetes (HR: 3.28, 95% CI: 2.30–4.67) compared with MHN adults (9), which was similar to the effect size in the Western population, but also lower than that in Chinese adults. Additionally, our study based on large-scale prospective research demonstrated that the HRs for T2DM in MHO phenotypes were relatively more significant in females than males. Together with previous literature reports, our findings do not support the notion that healthy obesity is a harmless condition, indicating that the current definitions of metabolic health are insufficient to identify a subgroup of obesity that is not at risk of T2DM (26, 27).

It is worth noting that we used Chinese-specific BMI and waist circumference, which is different from Western standards. Previous studies proved that Asian populations at a lower BMI than White populations have a high incidence rate of type 2 diabetes (28). Asian participants are more likely to have been allocated to the normal BMI group if using the same BMI cut-off points for all races. Hence, WHO recommended lower BMI cut-offs for defining obesity in south Asian populations to optimize the early identification of cardiometabolic risk. To better demonstrate the contrast between different BMI cut-off points in different people, some studies had included different ethnic groups to observe the relationship between different BMI cut-off points and glucose factors or the onset of diabetes after adjusting the same covariates (29, 30).

Although the MHO group had a higher risk of diabetes than the MHN, as reported previously (11), their risk was significantly lower than that in the MUO (12). At the same time, individuals with MUN had a higher risk of diabetes than MHN, indicating that a normal weight may not mean a healthy metabolic status. That is, normal-weight individuals may also be metabolically unhealthy (31). The potential mechanism is that metabolic risk in normal-weight subjects may be more strongly linked to a relatively low leg fat mass than high s.c. abdominal fat (32). This MUN phenotype also exhibits impaired insulin secretion capacity and insulin resistance, insufficient cardio-respiratory fitness, and increased carotid intima-media thickness (cIMT). In addition, genetic pathways of metabolic risk appear to be different between normal-weight and obese individuals (33, 34). It is necessary to follow the medical guideline-based recommendations, including the adoption of a healthy lifestyle and the initiation of pharmacological treatments for metabolic risk factors. Since fat mass and fat distribution differ between normal-weight and obese subjects, it is important to take targeted measures promoting fat transformation to reduce metabolic diseases risks (35, 36).

In addition, the effect on diabetes differs when a person is only obese or metabolically unhealthy. MHO individuals were at an increased risk of incident diabetes compared with MUN individuals with East Asian studies (9) but not with Western research results (12). For example, the Whitehall II cohort study among 7122 participants showed that MHO and MUN groups were at a similar increased risk of incident type 2 diabetes (12). The discrepancy of such association may be due to ethnic differences and the much smaller sample size but longer follow-up in the Western study.

Metabolic health might be a transient state for most obese individuals (15, 16, 37). In contrast to previous studies, which only examined the association of metabolic health at baseline with the risk of diabetes (9, 10, 13, 14), we addressed transition in obesity or metabolic health status over time by reviewing the health status at baseline and resurvey. Most metabolically healthy participants (67.74%) still maintain normal weight; only a small proportion (15.26%) of participants with initial MHN converted to MHOO after 10 years of follow-up. Similarly, people with overweight or obesity have a greater tendency to transit from metabolically healthy to metabolically unhealthy states. Whitehall II cohort study showed that about half of the participants with initial MHO converted to MUO over 20 years (38) which was somewhat higher than our study in Chinese adults (39.53%). The Nurses’ Health Study among 90 257 women indicated that only 6.1% of MHO were still metabolically healthy over 30 years (15). All participants were women, and a longer follow-up duration in the Nurses’ Health Study could explain the observed higher conversion rates (15).

However, no study investigated the association of the transition of individuals’ metabolic health with the risk of diabetes over a longer follow-up to our knowledge. In the present study, the high conversion rates (39.47%) from metabolic healthy to unhealthy obesity indicate that single-point determination of metabolic health is probably not appropriate to establish long-term diabetes risk precisely. Significantly, overweight or obese participates with stable metabolically unhealthy status showed a substantially higher risk of developing diabetes, which was two-fold higher than overweight or obese participants who changed from metabolically healthy to unhealthy situations. Based on the current study results, metabolic healthy obese alone cannot always be regarded as a stable characterization of lower clinical risk. Yet, it’s worth noting that only around 5% of the baseline studied population had measurements at the second resurvey. It is a fact that the existence of sampling error is inevitable. Hence, we conclude that the prevalence of metabolic status needs to be reviewed critically. Future studies should pay more attention to the transition of metabolic health and its increased risk for diabetes.

The strengths of the current study include the large number of diabetic cases, the prospective design, the repeated measurements, its long follow-up time, the high quality of cardiometabolic risk factors measurement, and the implementation of different sensitivity analyses. Further, all participants were recruited at random and consecutively from the general population; thus, the potential for selection bias is minimal. Notably, we considered the transition of metabolic health over time in estimating the risk of diabetes specifically among individuals with obesity (15, 39); therefore, the accuracy of diabetes risk estimates might have been improved. For our observational studies, however, the results must be interpreted with caution. There are also some limitations that should be mentioned. First, as the study population consisted of Chinese adults, the generalizability to other ethnic groups is limited. Secondly, similar to the definition of metabolic health in the previous study (26), we considered anti-dyslipidemia drugs in defining metabolic status, which may not rule out that stricter definitions of metabolic health components would identify a healthier subgroup. Thirdly, although we validated our results in resurvey data, misclassification of metabolic health which might bias the observed associations cannot be excluded. Fourthly, we adjusted for cardiometabolic risk factors, but residual confounding remains possible as generally in observational studies. Fifthly, as diabetes was the outcome of interest, we further excluded participants with missing baseline glucose data to minimize potential reverse causality bias for those who might have undiagnosed diabetes. The incidence density of diabetes and diabetes mortality was higher among participants with missing glucose data (Supplementary data and Supplementary Table 1). This strict exclusion criterion may have introduced selection bias, leading to underestimation of diabetes and diabetes mortality incidence rate.

In conclusion, overweight or obese participants with and without metabolic syndrome are associated with an increased risk of diabetes in the general population. Our results also reinforce recommendations that maintaining metabolic health in all BMI groups for diabetes prevention may be helpful in China. Moreover, metabolically healthy status as a transient state was shown to far increase the risk of type 2 diabetes when it changed to a metabolically unhealthy situation. Particular attention should be given to the impact of metabolic health and how it changes over time increasing the risk of diabetes risk.

## Supplementary Material

Supplementary Figure 1

Supplementary Figure 2

Supplementary Figure 3

eTable 1 Baseline characteristics of participants with and missing plasma glucose

eTable 2 Frequency distribution of metabolically healthy obesity at baseline and 2<sup>nd</sup> resurvey

eTable 3 Sensitivity analyses of adjusted hazard ratios for diabetes by MHO at baseline

eTable 4 Subdistribution hazard ratios from Fine-Gray regression models for diabetes by MHO at baseline

eTable 5 Adjusted odds ratios for diabetes by MHO status change from baseline to the 2<sup>nd</sup> resurvey

## Declaration of interest

All authors declare no support from companies for the submitted work; no relationships with companies that might have an interest in the submitted work in the previous 3 years; no spouses, partners, or children that have financial relationships that may be relevant to the submitted work; and no non-financial interests that may be relevant to the submitted work.

## Funding

This work was supported by grants (2020YFC2003401, 2016YFC0900500, 2016YFC0900501, 2016YFC0900504) from the National Key R&D Program of China. The CKB survey was supported by grants from the Kadoorie Charitable Foundation in Hong Kong, National Natural Science Foundation of China (81390540, 81390544, 81390541), and the Chinese Ministry of Science and Technology (2011BAI09B01). Funders have no role in research design, data collection, data analysis and interpretation, report writing or submission decisions for publication.

## Availability of data and material

Data are available in a public, open access repository. This research has been conducted using the China Biobank Resource under Application Number 2018-0066. The China Biobank data are available on application to the China Biobank (https://www.ckbiobank.org/site/).

## Ethics approval

The Ethical Review Committee of the Chinese Center for Disease Control and Prevention (Beijing, China) and the Oxford Tropical Research Ethics Committee, University of Oxford (UK), approved the study.

## Consent to participate

All participants consent to participate in the present study.

## Consent for publication

Consent for publication was obtained from all authors.

## Author contribution statement

Conceptualization was done by Canqing Yu, Yiping Chen, Zhengming Chen, Tao Huang, Liming Li. Data curation was done by Zimin Song, Meng Gao, Jun Lv, Canqing Yu, Yu Guo, Zheng Bian, Yuxia Wei, Huaidong Du, Ling Yang, Zhengming Chen, Liming Li. Formal analysis was done by Yu Guo, Yuxia Wei, Huaidong Du, Ling Yang, Yiping Chen, Jianqiang Zhang, Jvying Yao, Junshi Chen, Tao Huang, Liming Li. Funding acquisition was done by Liming Li. Investigation was done by Zhengming Chen, Tao Huang, Liming Li. Zimin Song, Meng Gao, Jun Lv, Zheng Bian, Yuxia Wei, Zhengming Chen provided the methodology. The original draft was written by Zimin Song, Meng Gao, Yu Guo, Zheng Bian, Yuxia Wei, Huaidong Du, Ling Yang, Yiping Chen, Jianqiang Zhang, Jvying Yao, Junshi Chen, Zhengming Chen, Tao Huang, Liming Li. The writing was reviewd and edited by Tao Huang, Liming Li.
