# Supplementary figures and images for: Metabolically healthy obesity, transition to unhealthy phenotypes, and type 2 diabetes in 0.5 million Chinese adults: the China Kadoorie Biobank
Source: Eur J Endocrinol. 2021 Dec 7;186(2):233–44. doi: 10.1530/EJE-21-0743 (PMC8789025; doi:10.1530/EJE-21-0743)

(A)

Baseline

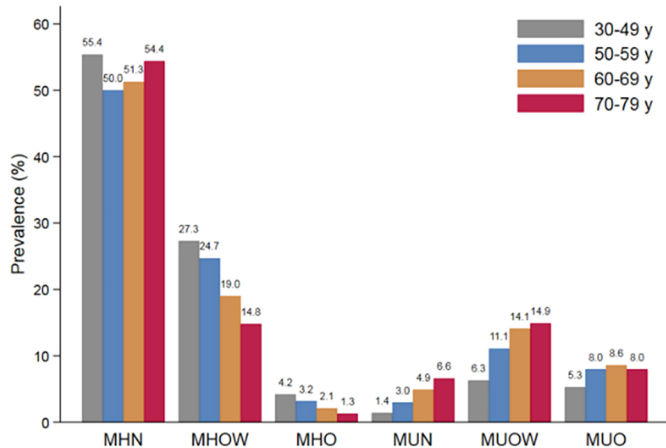

(B)

The second re-survey

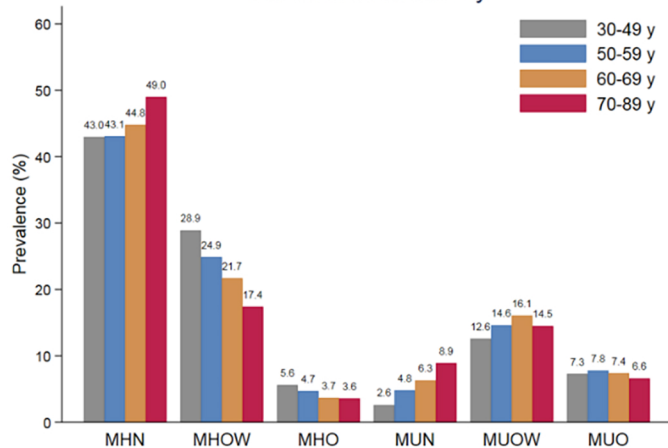

Supplement: Supplementary Figure 1 [file supplementary_figure_1.pdf]

(A)

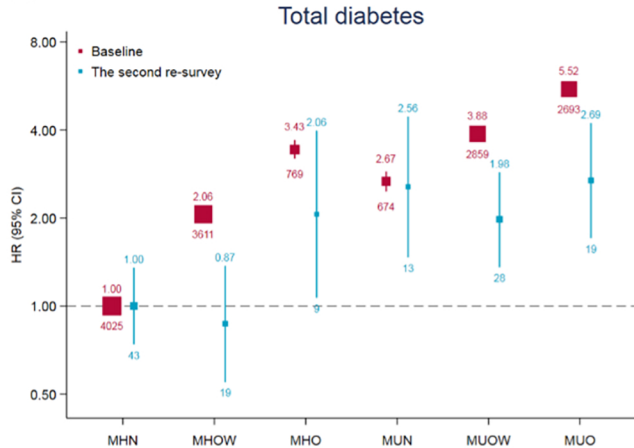

(B)

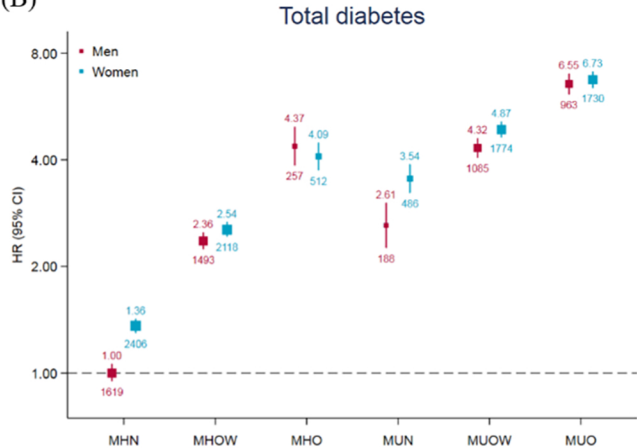

Supplement: Supplementary Figure 2 [file supplementary_figure_2.pdf]

(A)

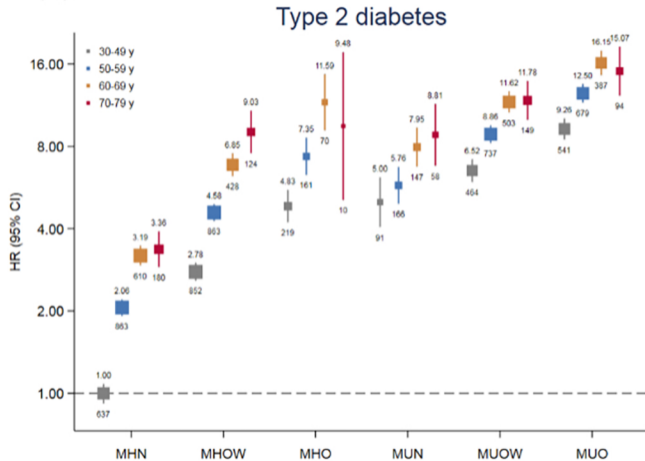

(B)

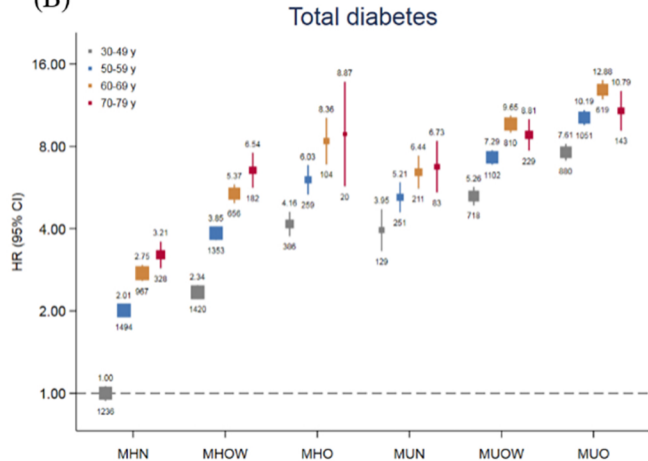

Supplement: Supplementary Figure 3 [file supplementary_figure_3.pdf]
