# Supplementary material for: Metabolically healthy obesity, transition to unhealthy phenotypes, and type 2 diabetes in 0.5 million Chinese adults: the China Kadoorie Biobank
Source: Eur J Endocrinol. 2021 Dec 7;186(2):233–44. doi: 10.1530/EJE-21-0743 (PMC8789025; doi:10.1530/EJE-21-0743)
Supplement: eTable 1 Baseline characteristics of participants with and missing plasma glucose [file supplementary_table_1.pdf]

eTable 1 Baseline characteristics of participants with and missing plasma glucose

Characteristics[†]	Participants missing plasma glucose (n=7479)	Participants with plasma glucose (n=432763)	P
**Demographic factors**			
Age (y)	50.1 (10.1)	51.0 (10.4)	<0.001
Male (%)	41	41.3	0.55
Urban (%)	6.1	43.5	<0.001
**Socioeconomic factors**			
Middle school or higher (%)	48.5	50.1	0.001
Household income≥20,000 yuan/year (%)	35.4	43.1	<0.001
Married (%)	91.2	91.4	0.620
**Lifestyle factors**			
Current smoker (%)	29.7	29.2	0.239
Weekly drinker (%)	17.1	15.4	<0.001
Physical activity (MET h/d)	22.9 (16.4)	21.9 (13.9)	<0.001
Meat intake (day/week)	3.8 (2.4)	3.7 (2.5)	0.103
Vegetable intake (day/week)	6.8 (0.8)	6.8 (0.8)	<0.001
Fruit intake (day/week)	3.2 (2.4)	2.6 (2.5)	<0.001
**Physical measurements**			
BMI (kg/m2)	23.6 (3.0)	23.8 (3.1)	<0.001
Waist hip ratio	0.9 (0.1)	0.9 (0.1)	<0.001
SBP (mmHg)	126.0 (20.5)	130.4 (20.8)	<0.001
DBP (mmHg)	76.0 (10.7)	77.8 (11.1)	<0.001
**Self-reported conditions (%)**			
High waist	24.4	23.3	0.035
Hypertension	41.1	48.8	<0.001
Hyperlipidemia	0.3	0.1	<0.001
**Outcome***			
Total diabetes	42.93 (39.20-46.66)	35.01 (34.44-35.58)	<0.001
Type 2 diabetes mellitus	24.95 (22.34-27.55)	21.56 (21.11-22.00)	0.007
Total diabetes mortality	1.20 (0.45-1.95)	0.48 (0.41-0.55)	0.005
Type 2 diabetes mellitus mortality	0.65 (0.10-1.20)	0.25 (0.20-0.30)	0.034

[†]All variables were adjusted for age, sex and region except for age and sex.

*Incidence density per 10000 person-years.

Abbreviations: MET-h/d, metabolic equivalents of task per hour per day; BMI, body mass index; SBP, systolic blood pressure; DBP, diastolic blood pressure
