# Supplementary material for: Metabolically healthy obesity, transition to unhealthy phenotypes, and type 2 diabetes in 0.5 million Chinese adults: the China Kadoorie Biobank
Source: Eur J Endocrinol. 2021 Dec 7;186(2):233–44. doi: 10.1530/EJE-21-0743 (PMC8789025; doi:10.1530/EJE-21-0743)
Supplement: eTable 2 Frequency distribution of metabolically healthy obesity at baseline and 2nd resurvey [file supplementary_table_2.pdf]

eTable 2 Frequency distribution of metabolically healthy obesity at baseline and 2nd resurvey

Baseline	2nd resurvey						Sum
	MHN	MHOW	MHO	MUN	MUOW	MUO	
MHN	6613	1485	5	883	743	34	9763
MHOW	567	2,205	290	132	1,502	402	5098
MHO	4	94	219	1	60	326	704
MUN	206	31	0	70	57	2	366
MUOW	89	344	65	76	665	222	1461
MUO	6	69	263	3	151	777	1269
Sum	7485	4228	842	1165	3178	1763	18661

Abbreviations: MHN, Metabolically healthy normal weight; MHOW, Metabolically healthy overweight; MHO, Metabolically healthy obesity; MUN, Metabolically unhealthy normal weight; MUOW, Metabolically unhealthy overweight; MUO, Metabolically unhealthy obesity. Sum, Summary.
