# Supplementary material for: Metabolically healthy obesity, transition to unhealthy phenotypes, and type 2 diabetes in 0.5 million Chinese adults: the China Kadoorie Biobank
Source: Eur J Endocrinol. 2021 Dec 7;186(2):233–44. doi: 10.1530/EJE-21-0743 (PMC8789025; doi:10.1530/EJE-21-0743)
Supplement: eTable 3 Sensitivity analyses of adjusted hazard ratios for diabetes by MHO at baseline [file supplementary_table_3.pdf]

eTable 3 Sensitivity analyses of adjusted hazard ratios for diabetes by MHO at baseline

	Baseline					
	MHN	MHOW	MHO	MUN	MUOW	MUO
**Total diabetes**						
Excluding participants who never smoke						
Cases	2417	2175	505	488	1827	1762
Person-years	1 377 062	685 260	107 917	76 956	241 446	186 484
HRs (95% CI)	1.00 (0.96-1.04)	1.89 (1.81-1.97)	3.05 (2.80-3.33)	2.64 (2.41-2.89)	3.66 (3.49-3.83)	5.06 (4.83-5.31)
Excluding cases within the first 2 years						
Cases	3628	3359	729	573	2550	2456
Person-years	2 276 058	1 063 180	150 325	110 590	384 645	280 872
HRs (95% CI)	1.00 (0.97-1.03)	2.11 (2.04-2.18)	3.55 (3.30-3.82)	2.59 (2.38-2.81)	3.86 (3.72-4.02)	5.56 (5.34-5.79)
Excluding waist circumference criterion						
Cases	1618	906	35	3081	5564	3427
Person-years	1 335 803	415 372	18 614	1 051 413	1 033 135	412 920
HRs (95% CI)	1.00 (0.95-1.05)	1.90 (1.78-2.03)	1.77 (1.27-2.46)	1.87 (1.80-1.94)	3.87 (3.76-3.97)	6.46 (6.25-6.68)
WHR instead of waist circumference						
Cases	2636	2031	709	2063	4439	2753
Person-years	1872186	759313	144774	515031	689194	286760
HRs (95% CI)	1.00 (0.96-1.04)	2.05 (1.96-2.14)	3.95 (3.67-4.26)	2.30 (2.20-2.41)	4.08 (3.96-4.20)	6.59 (6.35-6.84)
**Type 2 diabetes**						
Excluding participants who never smoke						
Cases	1365	1346	305	334	1205	1129
Person-years	1 381 868	688 818	108 629	77 872	244 221	188 994
HRs (95% CI)	1.00 (0.95-1.05)	2.14 (2.03-2.26)	3.51 (3.13-3.93)	2.94 (2.63-3.27)	4.20 (3.97-4.45)	5.87 (5.53-6.22)
Excluding cases within the first 2 years						
Cases	2116	2133	434	418	1689	1556
Person-years	2 283 946	1 068 814	151 485	111 811	389 157	284 878
HRs (95% CI)	1.00 (0.96-1.04)	2.42 (2.32-2.53)	4.05 (3.68-4.46)	2.98 (2.70-3.28)	4.48 (4.27-4.70)	6.46 (6.14-6.79)
Excluding waist circumference criterion						
Cases	836	521	19	1916	3599	2142
Person-years	1339305	416866	18677	1056683	1041468	417888
HRs (95% CI)	1.00 (0.93-1.07)	2.23 (2.04-2.43)	2.03 (1.29-3.18)	2.09 (1.99-2.19)	4.78 (4.63-4.94)	8.06 (7.72-8.41)
WHR instead of waist circumference						
Cases	1426	1152	398	1326	2968	1763
Person-years	1877669	762726	145921	518319	695608	290643
HRs (95% CI)	1.00 (0.95-1.05)	2.30 (2.17-2.44)	4.57 (4.14-5.04)	2.61 (2.47-2.76)	5.02 (4.84-5.21)	8.11 (7.74-8.50)

†HRs were adjusted for age (5years), sex, study region, educational level (primary school or lower, middle school or higher), household income (<20,000 yuan/year, or ≥20,000 yuan/year), marital status (married, others), smoking status (current regular smoker, not current regular smoker), alcohol consumption (weekly drinker, not weekly drinker), frequency of fruit intake, frequency of vegetable intake, frequency of meat intake (day/week), family history of diabetes and physical activity (3 groups).

Abbreviations: MHN, metabolically healthy normal weight; MHO, metabolically healthy obesity; MHOW, metabolically healthy overweight; MUN, metabolically unhealthy normal weight; MUO, metabolically unhealthy obesity; MUOW, metabolically unhealthy overweight; HR, hazard ratio; BMI, body mass index; CI, confidence interva
