# Supplementary material for: Metabolically healthy obesity, transition to unhealthy phenotypes, and type 2 diabetes in 0.5 million Chinese adults: the China Kadoorie Biobank
Source: Eur J Endocrinol. 2021 Dec 7;186(2):233–44. doi: 10.1530/EJE-21-0743 (PMC8789025; doi:10.1530/EJE-21-0743)
Supplement: eTable 4 Subdistribution hazard ratios from Fine-Gray regression models for diabetes by MHO at baseline [file supplementary_table_4.pdf]

**eTable 4 Subdistribution hazard ratios from Fine-Gray regression models for diabetes by MHO at baseline**

| | Baseline | | | | | | |
|---|---|---|---|---|---|---|---|
| | MHN | MHOW | MHO | MUN | MUOW | MUO | p-value |
| **Total diabetes death** | | | | | | | |
| Cases | 85 | 33 | 4 | 10 | 36 | 28 | <0.001 |
| Person-years | 2292009 | 1077174 | 153062 | 113775 | 396497 | 291649 | |
| subHR, multivariable-adjusted | 1.00 (ref) | 1.28 (0.85-1.92) | 1.53 (0.56-4.19) | 1.76 (0.89-3.46) | 2.26 (1.52-3.37) | 3.32 (2.13-5.18) | |
| **Type 2 diabetes death** | | | | | | | |
| Cases | 45 | 19 | 2 | 4 | 19 | 15 | <0.001 |
| Person-years | 2292009 | 1077174 | 153062 | 113775 | 396497 | 291649 | |
| subHR, multivariable-adjusted | 1.00 (ref) | 1.39 (0.80-2.42) | 1.48 (0.35-6.22) | 1.23 (0.44-3.43) | 2.25 (1.29-3.94) | 3.54 (1.89-6.60) | |
| **Other causes death** | | | | | | | |
| Cases (%) | 16642 (6.9%) | 4985 (5.8%) | 533 (5.7%) | 1196 (7.4%) | 3373 (6.7%) | 2120 (7.2%) | 0.165 |

†HRs were adjusted for sex, age (5 years), study region, educational level (primary school or lower, middle school or higher), household income (<20,000 yuan/year, or ≥20,000 yuan/year), marital status (married, others), smoking status (current regular smoker, not current regular smoker), alcohol consumption (weekly drinker, not weekly drinker), frequency of fruit intake, frequency of vegetable intake, frequency of meat intake (day/week), family history of diabetes and physical activity (3 groups).

Abbreviations: MHN, metabolically healthy normal weight; MHO, metabolically healthy obesity; MHOW, metabolically healthy overweight; MUN, metabolically unhealthy normal weight; MUO, metabolically unhealthy obesity; MUOW, metabolically unhealthy overweight; HR, hazard ratio
