# Supplementary material for: Metabolically healthy obesity, transition to unhealthy phenotypes, and type 2 diabetes in 0.5 million Chinese adults: the China Kadoorie Biobank
Source: Eur J Endocrinol. 2021 Dec 7;186(2):233–44. doi: 10.1530/EJE-21-0743 (PMC8789025; doi:10.1530/EJE-21-0743)
Supplement: eTable 5 Adjusted odds ratios for diabetes by MHO status change from baseline to the 2nd resurvey [file supplementary_table_5.pdf]

eTable 5 Adjusted odds ratios for diabetes by MHO status change from baseline to the 2nd resurvey

	Baseline				
	Stable MHN	MHN to MHOO	Stable MHOO	MHOO to MUOO	Stable MUOO
**Total diabetes**					
Cases	6613	1490	2808	2290	1815
OR, sex-adjusted	1.00 (ref)	0.76 (0.46-1.26)	1.15 (0.83-1.59)	4.28 (3.33-5.49)	8.66 (6.85-10.95)
OR, multivariable-adjusted	1.00 (ref)	0.78 (0.47-1.28)	1.15 (0.83-1.60)	4.25 (3.30-5.47)	8.60 (6.78-10.91)
**Type 2 diabetes**					
OR, sex-adjusted	1.00 (ref)	0.89 (0.48-1.66)	0.99 (0.64-1.54)	4.20 (3.03-5.81)	10.79 (8.04-14.48)
OR, multivariable-adjusted	1.00 (ref)	0.91 (0.49-1.68)	1.00 (0.64-1.56)	4.18 (3.01-5.81)	10.85 (8.04-14.63)

†Sex-adjusted model: Results were adjusted for sex, age (5 years), and study region.

‡Multivariable-adjusted model: Results were adjusted for sex, age (5 years), study region, educational level (primary school or lower, middle school or higher), household income (<20,000 yuan/year, or ≥20,000 yuan/year), marital status (married, others), smoking status (current regular smoker, not current regular smoker), alcohol consumption (weekly drinker, not weekly drinker), frequency of fruit intake, frequency of vegetable intake, frequency of meat intake (day/week), family history of diabetes and physical activity (3 groups).

Abbreviations: MHN, Metabolically healthy normal weight; MHOO, Metabolically healthy overweight or obesity; MUOO, Metabolically unhealthy overweight or obesity
